# Serogroup W-135 Meningococcal Disease during the Hajj, 2000

**DOI:** 10.3201/eid0906.020565

**Published:** 2003-06

**Authors:** Jairam R. Lingappa, Abdullah M. Al-Rabeah, Rana Hajjeh, Tajammal Mustafa, Adel Fatani, Tami Al-Bassam, Amira Badukhan, Abdulhafiz Turkistani, Nassen Al-Hamdan, Mohamed Al-Jeffri, Yaqoub Al Mazrou, Bradley A. Perkins, Tanja Popovic, Leonard W. Mayer, Nancy E. Rosenstein

**Affiliations:** * Centers for Disease Control and Prevention, Atlanta, Georgia, USA; † Saudi Arabian Ministry of Health, Riyadh, Kingdom of Saudi Arabia

**Keywords:** Meningococcal infections, meningitis, meningococcal, Neisseria meningitides, epidemiology, disease outbreaks, Saudi Arabia, Africa, research

## Abstract

An outbreak of serogroup W-135 meningococcal disease occurred during the 2000 Hajj in Saudi Arabia. Disease was reported worldwide in Hajj pilgrims and their close contacts; however, most cases were identified in Saudi Arabia. Trends in Saudi meningococcal disease were evaluated and the epidemiology of Saudi cases from this outbreak described. Saudi national meningococcal disease incidence data for 1990 to 2000 were reviewed; cases from January 24 to June 5, 2000 were retrospectively reviewed. The 2000 Hajj outbreak consisted of distinct serogroup A and serogroup W-135 outbreaks. Of 253 identified cases in Saudi Arabia, 161 (64%) had serogroup identification; serogroups W-135 and A caused 93 (37%) and 60 (24%) cases with attack rates of 9 and 6 cases per 100,000 population, respectively. The 2000 Hajj outbreak was the first large serogroup W-135 meningococcal disease outbreak identified worldwide. Enhanced surveillance for serogroup W-135, especially in Africa, is essential to control this emerging epidemic disease.

Meningococcal disease, caused by the bacterium *Neisseria meningitidis*, results in meningitis and sepsis in persons of all ages. The disease has a case-fatality rate of at least 10%, and chronic sequelae occur in 12% to 15% of survivors (*1*). Among the 13 meningococcal serogroups defined by serologic reactivity of the meningococcal capsular polysaccharide, serogroups A, B, and C are most commonly associated with disease worldwide, while other serogroups (e.g., X and Y) are of increasing importance (*1*,*2*). Although most meningococcal disease is sporadic, outbreaks also occur, historically caused by serogroups A, B, and C (*1*). Outbreaks can vary in magnitude, depending on many factors, including the serogroup and molecular characteristics of the epidemic strain. Serogroup A outbreaks in the “meningitis belt” of sub-Saharan Africa reach incidence rates of hundreds of cases per 100,000 population during a single dry season (*3*–*6*); serogroup B outbreaks are characterized by increased rates of disease occurring over years (*7*–*9*); and serogroup C outbreaks usually involve smaller numbers of cases occurring over weeks to months (*10*,*11*). Molecular characterization of *N. meningitidis* isolates has identified major electrophoretic-type (ET) complexes, such as ET-5 (*7*–*9*), and the ET-37 complex (*12*), as primarily associated with outbreaks of serogroups B and C disease, respectively.

In 1987, a meningococcal outbreak was associated with the Hajj pilgrimage (*13*). This pilgrimage, one of the central religious duties of Islam, draws 1–2 million Muslims from around the world to Saudi Arabia. The 1987 outbreak was caused by a strain of *N. meningitidis* serogroup A termed subgroup III. This subgroup was first associated with an outbreak in Nepal in 1983 and 1984 and after the 1987 Hajj outbreak caused massive meningococcal outbreaks in sub-Saharan Africa (*4*,*14*) with incidence rates from 250 to 1,000 cases/100,000 persons and at-risk populations numbering in the millions (*5*,*6*).

In response to the 1987 outbreak, Saudi Arabia required proof of meningococcal vaccination to issue Hajj pilgrimage visas (*15*). Bivalent (serogroup A+C) polysaccharide meningococcal vaccine is commonly used to meet this requirement (*16*); however, a quadrivalent serogroup A/C/Y/W-135 vaccine is used in some countries, particularly the United States (*17*). Vaccination campaigns with bivalent meningococcal vaccine have also been the principal strategy for controlling serogroup A outbreaks in Africa (*18*,*19*).

During the 2000 Hajj, an outbreak of meningococcal disease principally involved serogroup W-135 (17,20,21), an uncommon cause of disease that accounts for <2% of cases worldwide (*2*). Cases were reported in returning pilgrims in countries throughout the world including Europe, the United States, Asia, Africa, and the Middle East (*17*,*21*,*22*); however, most cases were identified in Saudi Arabia. The carriage and transmission characteristics of serogroup W-135 are not well understood. Furthermore, bivalent meningococcal vaccine cannot protect against serogroup W-135 disease. Therefore, if serogroup W-135 causes large outbreaks in Africa, as subgroup III did during the last decade, the practical implications will be serious. We evaluated recent trends of meningococcal disease in Saudi Arabia and examined demographic and clinical characteristics of the 2000 Hajj meningococcal cases in Saudi Arabia to assess the global impact of serogroup W-135 outbreaks.

## Materials and Methods

### Surveillance

Since 1990, the Saudi Arabian Ministry of Health has conducted national surveillance for meningococcal disease. From 1995 through 2000, the Ministry of Health collected data for the number of cases by serogroup and month; for 1990 through 1995, only national serogroup-specific totals by year were available.

During the 5-day Hajj pilgrimage, pilgrims complete a 24-mile round-trip journey from Mecca through the Plain of Arafat; many pilgrims subsequently perform additional ritual activities in Medina. During the 2 weeks before and 1 week after Hajj, Ministry of Health officials move meningococcal surveillance operations to Mecca and coordinate with regional teams conducting active surveillance for meningitis through daily contact with local hospitals, laboratories, and public health personnel in Mecca, Medina, and Jeddah. Surveillance data include demographic information, results of blood and cerebrospinal fluid (CSF) culture, serogroup identification of meningococcal isolates, and latex agglutination of CSF. Since most cases of meningococcal disease in the 2000 outbreak were reported in Mecca and Medina and since most Hajj pilgrims fly into the country through Jeddah, we focused our outbreak investigation on these three cities. The timing of the pilgrimage is based on the Islamic lunar calendar; the 2000 Hajj pilgrimage was March 14–19. We reviewed records for suspected meningococcal disease cases (see definition below) identified in Mecca, Medina, and Jeddah from January 21 through June 5, 2000. Demographic, microbiologic, and clinical data were collected from records compiled by the Ministry of Health and regional health directorates and through review of clinical laboratory records and inpatient charts from all hospitals serving the three regions.

Eighty-nine cases of serogroup W-135 disease were also reported to the World Health Organization from countries outside Saudi Arabia in association with pilgrims returning from the 2000 Hajj (*22*,*23*); additional information on these cases was not collected.

### Definitions

A case of meningitis was suspected for patients with fever (rectal temperature _l_>38.5°C or axillary temperature >38.0°C) and stiff neck (or bulging fontanel for patients <1 year of age). Confirmed meningococcal disease was defined as culture of *N. meningitidis* from blood or CSF, or detection of *N. meningitidis* antigen by latex agglutination of CSF from a person with suspected meningococcal disease in Mecca, Medina, or Jeddah from January 21 to June 5, 2000. Meningococcal meningitis was defined as a case of confirmed meningococcal disease with evidence for *N. meningitidis* in CSF through culture or latex agglutination; CSF specimens were evaluated for all cases reported here.

A patient was identified as a Saudi Arabian resident (Saudi or non-Saudi citizenship) if medical or surveillance records indicated that he or she resided in Saudi Arabia. Patients were defined as Hajj travelers if Ministry of Health or medical records indicated they had officially entered the country to perform Hajj or Umrah (a pilgrimage to Mecca that occurs year-round but is frequently performed by Hajj pilgrims just before Hajj); visitors without clear documentation were categorized as nonofficial. We defined Hajj pilgrims to include all persons who attended Hajj, based on Ministry of Health statistics. Severe illness was defined as admission to intensive care or death; chronic illness denoted a history of diabetes, hypertension, or heart disease. Meningococcal vaccination during the previous 3 years was recorded on Saudi Ministry of Health case report forms, which were typically obtained from the patient; vaccination status was not confirmed.

### Laboratory Analysis

*N. meningitidis* was identified on the basis of World Health Organization recommendations (*24*). Meningococcal serogroup identification was performed in the hospital clinical laboratory by latex agglutination for specific polysaccharide capsular types (A, B, C, Y, and W-135) on all available isolates with Wellcogen bacterial antigen kits (Murex Diagnostics Ltd., Dartford, U.K.); isolates were not retained in long-term storage. Cases without serogroup identification were categorized as serogroup unknown; this classification most commonly arose from lack of a viable isolate or unavailability of serogroup analysis kits. When the serogroup identified in case records conflicted with laboratory records, the serogroup identified in the hospital clinical laboratory log was used. Two serogroup W-135 and two serogroup A isolates collected from patients in Saudi Arabia were molecularly characterized by multilocus enzyme electrophoresis (MLEE) (*25*), serotyping/serosubtyping (*26*), pulsed-field gel electrophoresis (PFGE) (*27*), and multilocus sequence typing (*28*). Complete molecular subtyping of all serogroup W-135 isolates collected during the 2000 Hajj outbreak from patients identified in Saudi Arabia and from returning pilgrims whose illness was diagnosed in their country of origin are reported elsewhere (*29*). No serogroup W-135 isolates from Saudi cases identified before the 2000 Hajj were available for molecular characterization.

### Attack Rate Calculations

We calculated serogroup A and serogroup W-135 attack rates by using the official Saudi Ministry of Health estimate of 1.7 million Hajj attendees (*30*) as the denominator; the number of Hajj attendees by nationality was not available. For the serogroup W-135 attack rate, we combined the number of laboratory-confirmed serogroup W-135 cases with the estimated number of meningococcal disease cases of unknown serogroup attributed to serogroup W-135. The latter figure was estimated by starting with the total number of cases in which serogroup was identified, calculating the proportion of these attributed to serogroup W-135, and multiplying this proportion by the total number of cases in which serogroup was not identified. A similar procedure was used to calculate the serogroup A attack rate. Calculations, which were based on the serogroup distribution by region or for the outbreak as a whole, yielded similar results. We could not assess Hajj attendance of Saudi residents, and some patients may have been residents who did not attend Hajj; therefore, these calculations may overestimate the attack rate. The estimated 90 patients with serogroup W-135 disease identified in other countries associated with the 2000 Hajj (*22*,*23*) were not included in this analysis because of the lack of information identifying which cases arose through secondary transmission in the pilgrim’s country of origin after the Hajj.

### Statistical Analysis

Univariate analysis of the retrospective surveillance data was performed in Epi6 and EpiInfo 2000 (CDC, Atlanta, GA) and SAS 8.1 for Windows (SAS, Inc., Cary, NC) with the Fisher exact test with Mantel-Haenszel odds ratios (OR) to assess the association of individual variables with W-135 disease or severe illness. Demographic characteristics of cases with known versus unknown serogroup differed only in the high proportion of unknown serogroup cases identified in Jeddah. The category of unknown serogroup was treated as a missing value for all statistical analysis involving serogroup-specific data except attack rate calculations. Given the small number of cases in Jeddah and the similar serogroup-specific case distribution in Mecca, the variable identifying the metropolitan region where the case was diagnosed was divided into Medina versus Jeddah and Mecca.

All variables with p<0.1 on univariate analysis, as well as potential confounders, were entered into logistic regression. Stepwise, multivariate logistic regression was performed to assess risk factors associated with developing serogroup W-135 compared to either serogroup A disease or to all cases with known serogroup. Separate multivariable models were used to evaluate risk factors for severe illness and fatal disease.

## Results

**Figure 1:**
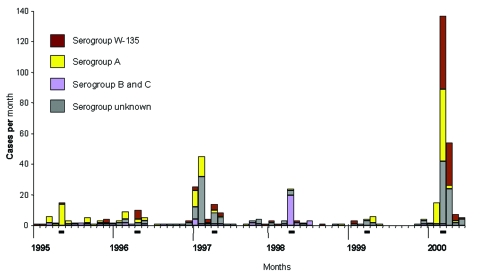
Meningococcal disease in Kingdom of Saudi Arabia, by month, 1995–2000. Cases of meningococcal disease with dates converted from Islamic calendar months. The period of the Hajj pilgrimage for each year is underscored.

From 1995 through 1999, the median annual number of meningococcal disease cases in Saudi Arabia (population 20 million) was 42 (range 20–107) with a resulting national annual meningococcal disease incidence of 0.2 cases/100,000 population (Figure 1). From 1995 to 1998, a median of 10 cases (range 4–20) occurred within 1 month of the Hajj. Most cases were serogroup A or W-135 disease; however, in 1998 a cluster of 20 cases of serogroup B meningococcal disease was identified. In 1997, a total of 72 cases of meningococcal disease, predominantly serogroup A, were reported in association with Umrah before the Hajj.

Serogroup W-135 has been present to a notable degree in Saudi Arabia at least since 1990. During 1995 through 1999, serogroup W-135 disease accounted for 13% of all meningococcal disease in Saudi Arabia (Figure 1). Saudi serogroup-specific surveillance data for 1990 through 1994 are available only by Islamic calendar year and not by month; during this time, serogroup W-135 accounted for 1.5% to 54% of annual meningococcal disease with the peak (24 cases) occurring in 1990–1991.

**Figure 2:**
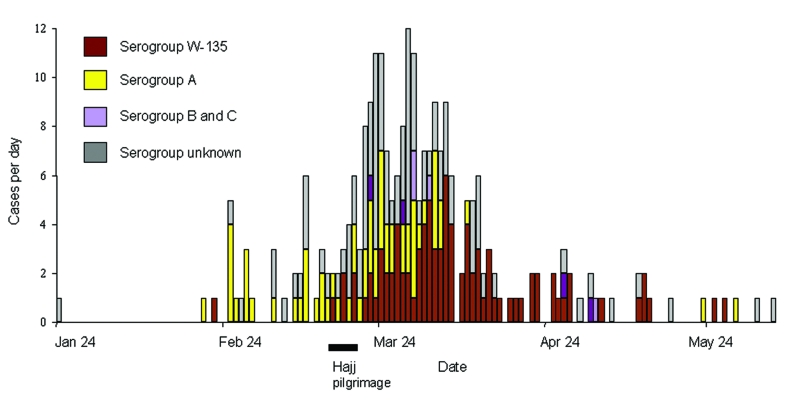
Meningococcal disease during the 2000 Hajj: Jeddah, Mecca, and Medina, January 24–June 5, 2000.  The number of cases of serogroup-specific meningococcal disease is shown by date. The duration of the 2000 Hajj is indicated.

In the year 2000, retrospective review identified 264 suspected cases of meningococcal disease in Mecca, Medina, or Jeddah. Of these, 253 (96%) were laboratory confirmed with 179 (71%) positive by CSF or blood culture and 74 (29%) negative by culture but positive by CSF latex agglutination. Seventy patients died, for a case-fatality rate of 28%. Cases increased during the month preceding the Hajj (Figure 2), peaking just after the Hajj ended; the last case was identified on June 5. The median age of patients with confirmed meningococcal disease was 40 years; most were male (Table 1). Overall, 56% of patients were Hajj travelers. Persons identified as Hajj travelers were older (median age 50 years vs. 5 years, p<0.001), more likely to be male (OR=1.9, p=0.02), and more likely to have a chronic illness (OR=7.8, p<0.001) than residents.

**Table 1:** Demographic characteristics of patients with confirmed meningococcal disease, Saudi Arabia^a^

Feature	All cases, n=253 (%)	Serogroup-specific cases, n (%)	
		W-135, n=93	A, n=60
Median date of hospital admission (range)	Mar 29 (Jan 24–June 2)	Apr 5 (Feb 22–May 24)	Mar 22(Feb 20–May 26)
Female^b^	118 (47)	48 (52)	20 (33)
Median age, y (range)	40 (0.2 to 80)	35 (0.2 to 80)	42 (2 to 75)
No. of cases identified in Jeddah	41 (16)	3 (3)	3 (5)
No. of cases identified in Mecca^b^	158 (62)	53 (57)	50 (83)
No. of cases identified in Medina^b^	54 (21)	37 (40)	7 (12)
**National status** ^b^			
Resident (Saudi/non-Saudi background)	98 (39)	43 (46)	17 (29)
Hajj or Umrah traveler	143 (57)	48 (52)	42 (71)
Nonofficial	11 (4)	2 (2)	0 (0)
Chronic illness	44 (17)	12 (13)	10 (17)
History of any meningococcal vaccination	130/222 (59)	55 (67)	30 (59)
**National origin**			
Africa	89 (35)	36 (39)	19 (32)
Asia^c^	106 (42)	31 (33)	29 (48)
Southwest Asia	65(26)	17(18)	23(38)
Europe	9 (4)	5 (5)	2 (3)
Middle East	48 (19)	21 (23)	10 (17)

^a^Identified by retrospective surveillance (January 24–June 5, 2000) in Jeddah, Mecca, and Medina. Cases of serogroup B and C disease (four cases each), as well as cases of unknown serogroup, are not shown. Denominator for all confirmed cases is 253 unless stated otherwise (changes in denominators are due to missing values); denominator for serogroup-specific percentages is the total serogroup-specific disease. ^b^ Comparison of characteristics for serogroup W-135 versus serogroup A with Fisher exact p value <0.05. ^d^Countries included in category of Southwest Asia: Afghanistan, Pakistan, India, Bangaladesh, Myanmar, and Sri Lanka.

A serogroup was identified for 161 (64%) of the 253 confirmed cases. Ninety-three cases (37% of all confirmed cases) were attributed to serogroup W-135 and 60 (24%) to serogroup A. Both serogroup A isolates from the 2000 outbreak were confirmed as subgroup III (ET-734, ST-5 with PFGE patterns characteristic for the “first pandemic wave” (*14*); serogroup W-135 isolates from the 2000 outbreak belonged to the ET-37 complex, as defined by MLEE, and were designated as the (W)ET-37 clone (*29*). Serogroup W-135 case-patients had a median date of admission 2 weeks later than for serogroup A patients (p<0.001) (Table 1). The diagnosis for most serogroup A patients was made in Mecca; serogroup W-135 cases were diagnosed in patients in both Mecca and Medina. Rates of meningococcal vaccination did not differ between serogroup W-135 and serogroup A case-patients (67% vs. 59%, respectively; p=0.35); however, among Hajj travelers, patients with serogroup W-135 were more likely to have received meningococcal vaccine than were those with serogroup A disease (93% vs. 77%, p=0.05). The calculated attack rates of serogroup W-135 and serogroup A disease were 8.9 and 5.8 cases/100,000, respectively. During the month when the outbreak peaked, weekly serogroup W-135 attack rates remained <1 case/100,000 persons. Multivariable modeling for risk factors for serogroup W-135 disease showed that, after age was controlled for, patients with cases diagnosed in Medina were at increased risk for serogroup W-135 compared with all other cases with known serogroups (OR=6, 95% confidence intervals [CI] 2.2 to 15.5, p<0.01). While no regional group was at increased risk for serogroup W-135 compared with serogroup A disease, serogroup A disease was more likely to occur in travelers arriving from Southwest Asia than serogroup W-135 disease (OR=2.6, 95% CI 1.1 to 5.6, p=0.02);

We also evaluated clinical characteristics of identified case-patients and found that approximately half were first seen by a clinician <1 day after symptom onset; at that time, 62% of case-patients were semicomatose, comatose, or dead. Only 9% of case-patients had sepsis without evidence of meningitis. Patients with serogroup W-135 compared with those with serogroup A were more likely not to have meningitis (OR 3.6, p=0.02) and to be admitted to intensive care (Table 2). Case-fatality rates did not differ by serogroup; patients with chronic illness had a higher case-fatality rate (50% vs. 19%, p=0.0003). For residents and Hajj travelers with serogroup W-135 disease, case-fatality rates were 12% and 46%, respectively (p<0.001). In a multivariate model controlling for age, factors associated with increased risk for fatal disease were the following: admission to intensive care (OR 15, 95% CI 5 to 45), chronic illness (OR 3, 95% CI 1 to 8), and being seen by a clinician <1 day of symptoms (OR 2.5, 95% CI 1 to 6); cases diagnosed in Medina were less likely to be fatal (OR=0.2, 95% CI 0.05 to 0.5). In this multivariate model, serogroup was not found to be a significant risk factor for fatal disease. A separate multivariate model for risk factors for severe illness, after age was controlled for, identified serogroup W-135 disease compared with all other cases of known serogroup (OR=2.0, 95% CI 1.2 to 5.2) and being seen by a clinician after <1 day of symptoms (OR=2.4, 95% CI 1.2 to 5.1) as associated with increased risk. In this model, persons with a history of chronic illness were at increased risk for severe illness, although this association did not reach statistical significance (OR 2.4, p=0.09).

**Table 2:** Clinical characteristics of patients with confirmed meningococcal disease, Saudi Arabia^a^

Feature	All cases, n=253 (%)	Serogroup-specific cases, n (%)	
		W-135, n=93	A, n=60
Seen by clinician <1 day after symptom onset	125 (49)	45 (48)	31 (52)
Semiconscious, comatose, or dead^b^	146 (62)	57 (63)	33 (58)
Positive blood culture^b^	72/140	55 (79)	15 (29)
Sepsis without meningitis	23 (9)	19 (20)	4 (7)
Admitted to intensive care^b^	51/212 (24)	27 (35)	7 (14)
>1-day intensive care required^b^	33/202 (16)	19 (26)	3 (6)
Death (case-fatality rate)	70 (28)	27 (29)	16 (27)

^a^Identified by retrospective surveillance (January 24–June 5, 2000, in Jeddah, Mecca, Medina, Saudi Arabia. Cases of serogroup B and C disease (four cases each) as well as cases of unknown serogroup are not shown. Denominator for all confirmed cases is 253 unless stated otherwise (changes in denominators are due to missing values); denominator for serogroup-specific percentages is the total serogroup-specific disease. ^b^Comparison of characteristics for serogroup W-135 versus serogroup A with Fisher exact p value <0.05.

## Discussion

The meningococcal outbreak during the 2000 Hajj was unusual because it consisted of two concurrent outbreaks, one caused by serogroup A and one caused by serogroup W-135. Both outbreaks were detected because the surveillance for meningococcal disease in Saudi Arabia, particularly during the Hajj, is extensive. In the African meningitis belt, serogroups are commonly identified on the basis of analysis of isolates early in the outbreak with little subsequent serogroup evaluation. If this practice had occurred during the 2000 Hajj outbreak, it would likely have been misidentified as an outbreak of solely serogroup A disease; the serogroup W-135 component would have been missed. Longitudinal serogroup-specific surveillance is especially essential for outbreak control with serogroup-specific vaccines.

Although isolates from Saudi Arabia before the 2000 outbreak were not available for analysis, other studies have found that the (W)ET-37 clone caused disease decades before the 2000 outbreak (*29*). Since serogroup W-135 disease appears to have been endemic in Saudi Arabia for at least the last decade, the (W)ET-37 outbreak strain was likely present in Saudi Arabia before the 2000 Hajj, and this outbreak was probably not due to introduction of a new clone. The (W)ET-37 clone probably not only caused invasive disease but also circulated in the population through nasopharyngeal carriage. In 2001, one study found rare serogroup W-135 carriage in returning pilgrims (*31*); another study found higher rates of carriage (*32*). However, the absence of carriage data from Saudi Arabia from 2000 or preceding years makes drawing conclusions about the complicated relationship between carriage and epidemic meningococcal disease in this setting difficult.

Despite previous circulation of the (W)ET-37 outbreak strain, the outbreak described here was the first large W-135 outbreak reported worldwide (*33*); the attack rate was 9 cases/100,000 population. Although the 2000 serogroup W-135 outbreak was larger than the 2000 serogroup A outbreak, this comparison must be put into the context of other outbreaks. During the 1987 Hajj, 1,841 laboratory-confirmed cases of serogroup A disease (*16*) were identified in Saudi Arabia among approximately 1.5 million Hajj attendees (*34*), for an incidence of 120/100,000. The *N. meningitidis* serogroup A isolates from the 2000 and 1987 Hajj outbreaks are members of the same serogroup A subgroup III, and yet the magnitude of the 2000 outbreak was less than one tenth that of the 1987 outbreak. The requirement for meningococcal vaccination was enacted in Saudi Arabia only after the 1987 Hajj. We found vaccination coverage in the 2000 outbreak case-patients in Saudi Arabia (59% to 67%) similar to coverage reported previously in Mecca residents (*16*); substantial blunting of serogroup A outbreaks elsewhere has been associated with similar levels of vaccination coverage (*5*,*18*). This finding suggests that the potential impact of the serogroup A outbreak during the 2000 Hajj may have also been markedly blunted by vaccination. However, since most vaccination for the Hajj is performed with bivalent, not quadrivalent, vaccine, vaccination coverage for serogroup W-135 during the 2000 Hajj was probably low (*35*). Therefore, the 2000 outbreak could be interpreted as representing the full outbreak potential of the (W)ET-37 clone within the context of the Hajj—much as the 1987 outbreak represented the unblunted impact of serogroup A subgroup III. Given that the 2000 serogroup W-135 outbreak was very small in comparison to the 1987 serogroup A outbreak, and weekly attack rates associated with the 2000 Hajj outbreak were substantially below the thresholds used to identify meningococcal outbreaks in Africa (*19*), within the context of the Hajj, the (W)ET-37 clone would appear to be less likely to cause outbreaks with incidence as high as those associated with serogroup A subgroup III. Clearly, this hypothesis is difficult to prove, since a constellation of host, environmental, and pathogen-specific factors in Mecca during the 2000 Hajj likely contributed to propagation of the outbreak strain; we were unable to identify the specific causative factors for this outbreak. A more recent (W)ET-37 outbreak in Burkina Faso, which had epidemiologic features typical of a serogroup A epidemic, may also have been caused by one or more of these factors (*36*).

Since 1987, a core component for preventing meningococcal disease during the Hajj has been mandatory vaccination of pilgrims and Saudi residents. In 2000, most vaccinated pilgrims received the bivalent vaccine. Given the large number of 2000 Hajj pilgrims and the estimated 85% to 95% vaccine efficacy against serogroup A disease (*37*,*38*) (and based on the assumption that reported Hajj pilgrim vaccinations are accurate), the 35 persons who reported having serogroup A disease despite having recently received meningococcal vaccine would be expected vaccine failures. Vaccine failure may have also led to the single primary case of serogroup W-135 disease (*39*) that occurred among the 11,000 Hajj pilgrims returning to the United States, where quadrivalent vaccine is used. In contrast, 10 primary cases of serogroup W-135 disease were reported among the 10,300 pilgrims returning to the United Kingdom, where bivalent vaccine is used (p=0.01) (*20*). Since no modifiable risk factors for disease have been identified for the 2000 serogroup W-135 outbreak, quadrivalent vaccine should be used to reduce the risk for serogroup W-135 disease. Given the high overall case-fatality rate of 28% for the 2000 Hajj outbreaks and our finding that chronic illness was a risk factor for severe illness and death, use of quadrivalent vaccine for older and chronically ill pilgrims is a priority. Strategies should also target this population for frequent evaluations for symptoms of disease.

Since 2002, Saudi Arabia has required quadrivalent vaccine for all pilgrims participating in the Hajj (*40*). However, the higher cost of this vaccine, as well as inadequate global vaccine supply, makes implementing this strategy difficult. If the (W)ET-37 clone continues to cause outbreaks in sub-Saharan Africa, as it did in Burkina Faso in 2002 (*36*), quadrivalent vaccine would be urgently needed for outbreak management. While empiric use of quadrivalent vaccine for outbreak response in Africa is not warranted at this time, heightened surveillance for serogroup W-135 disease, particularly in the Eastern Mediterranean region and the African meningitis belt, will be essential in designing and assessing future control and prevention strategies for this emergent epidemic disease.

## References

[1] Rosenstein NE, Perkins BA, Stephens DS, Popovic T, Hughes JM. Meningococcal disease. N Engl J Med. 2001;344:1378–88. 10.1056/NEJM200105033441807 11333996

[2] Rosenstein NE, Perkins BA, Stephens DS, Lefkowitz L, Cartter M, Danila R, The changing epidemiology of meningococcal disease in the United States, 1992–1996. J Infect Dis. 1999;180:1894–901. 10.1086/315158 10558946

[3] Lapeyssonnie L. La méningite cérébrospinale en Afrique. Bull World Health Organ. 1963;28:3–114. PMC2554630 14259333

[4] Moore PS, Reeves MW, Schwartz B, Gellin BG, Broome CV. Intercontinental spread of an epidemic group A *Neisseria meningitidis* strain. Lancet. 1989;2:260–3. 10.1016/S0140-6736(89)90439-X 2569063

[5] Pinner RW, Onyango F, Perkins BA, Mirza NB, Ngacha DM, Reeves M, Epidemic meningococcal disease in Nairobi, Kenya, 1989. The Kenya/Centers for Disease Control (CDC) Meningitis Study Group. J Infect Dis. 1992;166:359–64. 1634807 10.1093/infdis/166.2.359

[6] Greenwood B. Manson lecture. Meningococcal meningitis in Africa. Trans R Soc Trop Med Hyg. 1999;93:341–53. 10.1016/S0035-9203(99)90106-2 10674069

[7] Caugant DA, Froholm LO, Bovre K, Holten E, Frasch CE, Mocca LF, Intercontinental spread of *Neisseria meningitidis* clones of the ET-5 complex. Antonie van Leeuwenhoek. 1987;53:389–94. 10.1007/BF00415492 3130777

[8] Caugant DA. Population genetics and molecular epidemiology of *Neisseria meningitidis.* APMIS. 1998;106:505–25. 10.1111/j.1699-0463.1998.tb01379.x 9674888

[9] Fischer M, Perkins B. *Neisseria meningitidis* serogroup B: emergence of the ET-5 complex. Semin Pediatr Infect Dis. 1997;8:30–56. 10.1016/S1045-1870(97)80009-X

[10] Jackson LA, Schuchat A, Reeves MW, Wenger JD. Serogroup C meningococcal outbreaks in the United States. An emerging threat. JAMA. 1995;273:383–9. 10.1001/jama.273.5.383 7823383

[11] Centers for Disease Control and Prevention. Control and prevention of serogroup C meningococcal disease: evaluation and management of suspected outbreaks: recommendations of the Advisory Committee on Immunization Practices (ACIP). MMWR Morb Mortal Wkly Rep. 1997;46(RR-5):13–21. 9048847

[12] Wang JF, Caugant DA, Morelli G, Koumare B, Achtman M. Antigenic and epidemiologic properties of the ET-37 complex of *Neisseria meningitidis.* J Infect Dis. 1993;167:1320–9. 8501321 10.1093/infdis/167.6.1320

[13] Moore PS, Harrison LH, Teizak EE, Ajello GW, Broome CV. Group A meningococcal carriage in travelers returning from Saudi Arabia. JAMA. 1988;260:2686–9. 10.1001/jama.260.18.2686 3184335

[14] Zhu P, van der Ende A, Falush D, Brieske N, Morelli G, Linz B, Fit genotypes and escape variants of subgroup III *Neisseria meningitidis* during three pandemics of epidemic meningitis. Proc Natl Acad Sci U S A. 2001;98:5234–9. 10.1073/pnas.061386098 11287631 PMC33193

[15] Vaccination requirements. Pilgrimage to Mecca (Hajj). Wkly Epidemiol Rec. 1994;69:17. 8142247

[16] Al-gahtani YM, El Bushra HE, Al-Qarawi SM, Al-Zubaidi AA, Fontaine RE. Epidemiological investigation of an outbreak of meningococcal meningitis in Makkah (Mecca), Saudi Arabia, 1992. Epidemiol Infect. 1995;115:399–409. 10.1017/S0950268800058556 8557071 PMC2271597

[17] Centers for Disease Control and Prevention. Serogroup W-135 meningococcal disease among travelers returning from Saudi Arabia—United States, 2000. MMWR Morb Mortal Wkly Rep. 2000;49:345–6. 10817480

[18] Miller MA, Wenger J, Rosenstein N, Perkins B. Evaluation of meningococcal meningitis vaccination strategies for the meningitis belt in Africa. Pediatr Infect Dis J. 1999;18:1051–9. 10.1097/00006454-199912000-00005 10608623

[19] Detecting meningococcal meningitis epidemics in highly-endemic African countries. Wkly Epidemiol Rec. 2000;75:306–9. 11045076

[20] Samuelsson S, Handysides S, Ramsay M, Lyytikainen O, Nygard K, Perrocheau A, Meningococcal infection in pilgrims returning from the Hajj: update from Europe and beyond. Eurosurveillance Weekly. 2000;17:1–5.

[21] Taha MK, Achtman M, Alonso JM, Greenwood B, Ramsay M, Fox A, Serogroup W135 meningococcal disease in Hajj pilgrims. Lancet. 2000;356:2159. 10.1016/S0140-6736(00)03502-9 11191548

[22] Meningococcal disease, serogroup W-135. Wkly Epidemiol Rec. 2000;75:180.

[23] Aguilera JF, Perrocheau A, Meffre C, Hahne S. Outbreak of serogroup W135 meningococcal disease after the Hajj pilgrimage, Europe, 2000. Emerg Infect Dis. 2002;8:761–7. 12141959 10.3201/eid0808.010422 PMC2732506

[24] World Health Organization. Laboratory methods for the diagnosis of meningitis caused by *Neisseria meningitidis, Streptococcus pneumoniae*, and *Haemophilus influenzae*. Geneva. Organization. 1999; 1–74.

[25] Reeves MW, Perkins BA, Wenger JD. Epidemic-associated *Neisseria meningitidis* detected by multilocus enzyme electrophoresis. Emerg Infect Dis. 1995;1:53–4. 10.3201/eid0102.950203 8903159 PMC2626839

[26] Tondella ML, Popovic T, Rosenstein NE, Lake DB, Carlone GM, Mayer LW, Distribution of *Neisseria meningitidis* serogroup B serosubtypes and serotypes circulating in the United States. The Active Bacterial Core Surveillance Team. J Clin Microbiol. 2000;38:3323–8. 10970378 10.1128/jcm.38.9.3323-3328.2000 PMC87381

[27] Popovic T, Schmink S, Rosenstein NA, Ajello GW, Reeves MW, Plikaytis B, Evaluation of pulsed-field gel electrophoresis in epidemiological investigations of meningococcal disease outbreaks caused by *Neisseria meningitidis* serogroup C. J Clin Microbiol. 2001;39:75–85. 10.1128/JCM.39.1.75-85.2001 11136752 PMC87683

[28] Maiden MC, Bygraves JA, Feil E, Morelli G, Russell JE, Urwin R, Multilocus sequence typing: a portable approach to the identification of clones within populations of pathogenic microorganisms. Proc Natl Acad Sci U S A. 1998;95:3140–5. 10.1073/pnas.95.6.3140 9501229 PMC19708

[29] Mayer LW, Reeves MW, Al-Hamdan N, Sacchi CT, Taha MK, Ajello GW, Outbreak of W135 meningococcal disease in 2000: not emergence of a new W135 strain but clonal expansion within the electophoretic type-37 complex. J Infect Dis. 2002;185:1596–605. 10.1086/340414 12023765

[30] Saudi Arabian Ministry of Health. Number of pilgrims performing Hajj in 1420 AH. Riyadh: The Saudi Arabian Information Resource; 2000.

[31] Centers for Disease Control and Prevention. Public health dispatch: update: assessment of risk for meningococcal disease associated with the Hajj 2001. MMWR Morb Mortal Wkly Rep. 2001;50:221–2. 11300626

[32] Wilder-Smith A, Barkham TM, Ravindran S, Earnest A, Paton NI. Persistence of W135 *Neisseria meningitidis* carriage in returning Hajj pilgrims: risk for early and late transmission to household contacts. Emerg Infect Dis. 2003;9:123–6. 12533295 10.3201/eid0901.020131 PMC2873737

[33] Kwara A, Adegbola RA, Corrah PT, Weber M, Achtman M, Morelli G, Meningitis caused by a serogroup W135 clone of the ET-37 complex of *Neisseria meningitidis* in West Africa. Trop Med Int Health. 1998;3:742–6. 10.1046/j.1365-3156.1998.00300.x 9754670

[34] Saudi Arabia Ministry of Health. Annual health report, 1418 H. Riyadh (Saudi Arabia): The Ministry; 1997. p. 286.

[35] Noah N. *Neisseria meningitidis* W135: 2a: P1.2,5 arising from successive pilgrimages to Mecca. Eurosurveillance Weekly: Public Health Laboratory Service 2001;5(16).

[36] World Health Organization. Meningococcal disease, serogroup W135, Burkina Faso. Preliminary report, 2002. Wkly Epidemiol Rec. 2002;77:152–5. 12037945

[37] Artenstein M, Gold R, Zimmerly J, Wyle F, Schneider H, Harkins C. Prevention of meningococcal disease by group C polysaccharide vaccine. N Engl J Med. 1970;282:417–20. 10.1056/NEJM197002192820803 4983754

[38] Gold R, Artenstein M. Meningococcal infections: 2. Field trial of group C meningococcal polysaccharide vaccine in 1969-1970. Bull World Health Organ. 1971;45:279–82. 5316907 PMC2427931

[39] Centers for Disease Control and Prevention. Meningococcal disease among travelers returning from Saudi Arabia. MMWR Morb Mortal Wkly Rep. 1987;36:559. 3112548

[40] Meningococcal disease, serogroup W-135. Wkly Epidemiol Rec. 2001;76:141–2. 11383502

