# Correction, Vol. 9, No. 6

**DOI:** 10.3201/eid0908.C10908

**Published:** 2003-08

In “Serogroup W-135 Meningococcal Disease during the Hajj, 2000,” p. 665, author Sahar Makki was inadvertently not included. The correct author list should read as follows:

Jairam R. Lingappa,* Abdullah M. Al-Rabeah,† Rana Hajjeh,* Tajammal Mustafa,† Adel Fatani,† Tami Al-Bassam,† Amira Badukhan,† Abdulhafiz Turkistani,† Sahar Makki,† Nassen Al-Hamdan,† Mohamed Al-Jeffri,† Yaqoub Al Mazrou,† Bradley A. Perkins,* Tonja Popovic,* Leonard W. Mayer,* and Nancy E. Rosenstein*

*Centers for Disease Control and Prevention, Atlanta, Georgia, USA

††Saudi Arabian Ministry of Health, Riyadh, Kingdom of Saudi Arabia

